# ReferencesEfficiency and safety of renal denervation via cryoablation (Cryo-RDN) in Chinese patients with uncontrolled hypertension: study protocol for a randomized controlled trial

**DOI:** 10.1186/s13063-019-3693-9

**Published:** 2019-11-28

**Authors:** Han Chen, Meng Ji, Yi Zhang, Yawei Xu, Lingjuan Qiao, Li Shen, Junbo Ge

**Affiliations:** 1 Department of Cardiology, Zhongshan Hospital, Fudan University, Shanghai, China; 2 Institute of Biomedical Sciences, Fudan University, Shanghai, China; 3 Department of Cardiology, Shanghai Tenth People’s Hospital, Shanghai, China; 4 CryoFocus MedTech (Shanghai) Co., Ltd., Shanghai, China; 5 Shanghai Institute of Cardiovascular Diseases, Shanghai, China

**Keywords:** Renal denervation, Cryoablation, Hypertension

## Abstract

**Background:**

Clinical data show that due to the limited effects of lifestyle regulation and unsatisfactory drug adherence, only half of the hypertensive population have their blood pressure (BP) under control. In recent years, catheter-based renal denervation (RDN) has been used as a novel approach for treating uncontrolled hypertension. The safety and efficacy of catheter-based RDN have been confirmed by a number of studies and trials in which the participants were all non-Chinese and RDN was conducted via radiofrequency or ultrasound.

**Methods/design:**

This study is a prospective multicenter randomized sham-controlled trial that aims to investigate the safety and efficacy of cryoablation RDN (cryo-RDN) using a novel dedicated cryoablation balloon catheter (Cryofocus, China). A total of 200 Chinese patients who have uncontrolled hypertension despite standard medical treatment will be enrolled. With drug standardization, eligible participants will be randomized in a 1:1 ratio to undergo cryo-RDN treatment or renal angiography alone as a sham treatment.

The primary endpoint is defined as the change in 24-h ambulatory systolic blood pressure from baseline to 6 months. Office BP and other 24-h ambulatory BP are included as secondary endpoints. Safety endpoints primarily include any adverse effects.

**Discussion:**

This study was designed to verify the safety and efficacy of cryo-RDN with Cryofocus balloon catheters in uncontrolled hypertensive patients on polypharmacy. The aim is to provide a new way to improve the control of hypertension in China as a complement to drug therapy.

**Trial registration:**

ChiCTR, ChiCTR1800017707. Registered on 10 August 2018.

**Electronic supplementary material:**

The online version of this article (10.1186/s13063-019-3693-9) contains supplementary material, which is available to authorized users.

## Background

Globally, hypertension is a chronic disease and a leading risk factor for cardio- and cerebrovascular comorbidities, resulting in high mortality and economic burden [1, 2]. However, despite adequate pharmacological (drug combinations) and lifestyle changes, approximately 50% of the hypertensive population have suboptimal control of their blood pressure (BP) [3].

In 2009, Krum et al. provided the first report showing that renal denervation (RDN) was suitable for treating resistant hypertension [4]. In the last decade, there have been numerous RDN studies conducted with different designs [5, 6]. Although study outcomes vary, the safety of RDN in lowering BP is clear, but its efficacy was uncertain. However, after the positive results of the latest HTN MED-OFF trial, the efficacy of catheter-based RDN therapy is now well accepted [7].

Most RDN studies use a radiofrequency current and ultrasound as the energy sources for ablating the renal sympathetic nerves. In 2014, Prochnau et al. reported an ideal BP decline in 10 patients following cryoablation after a failed attempt at radiofrequency RDN [3]. Contrast agent in the balloon forms an ice ball, absorbing heat spherically from surrounding tissue.The overall freezing and rewarming procedure is much shorter than that for other denervation devices. Our previous animal study and the first-in-man study show that cryoablation RDN (cryo-RDN) is efficient and safe. There were no vascular events relating to BP reduction [8].

**Fig. 1:**
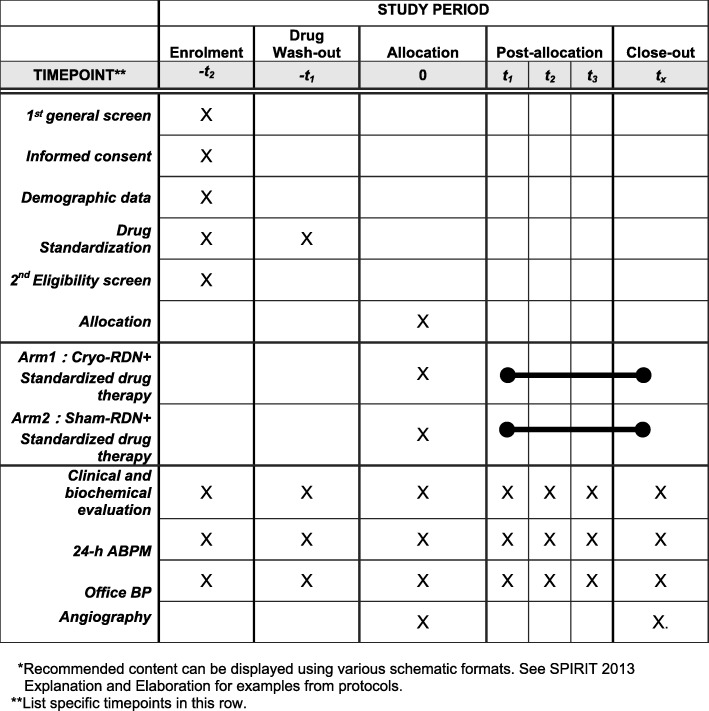
Schedule of enrolment, interventions, and assessments. Blood pressure monitor, cryo-RDN cryoablation renal denervation

In China, although approximately half of adults between the ages of 35 and 75 years have hypertension, many of these individuals have poorly controlled BP, with control rates and treatment control rates of 13.8% and 41.1%, respectively [9, 10]. To explore the safety and feasibility of cryo-RDN in Chinese patients with uncontrolled hypertension further, we have developed a rationale and methodology for a prospective multicenter randomized sham-controlled trial using a 7.5F cryoablation catheter from the Cryofocus system. Figure 1 is the schedule of enrolment, interventions, and assessments for the trial.

## Methods/design

### Study design

**Fig. 2:**
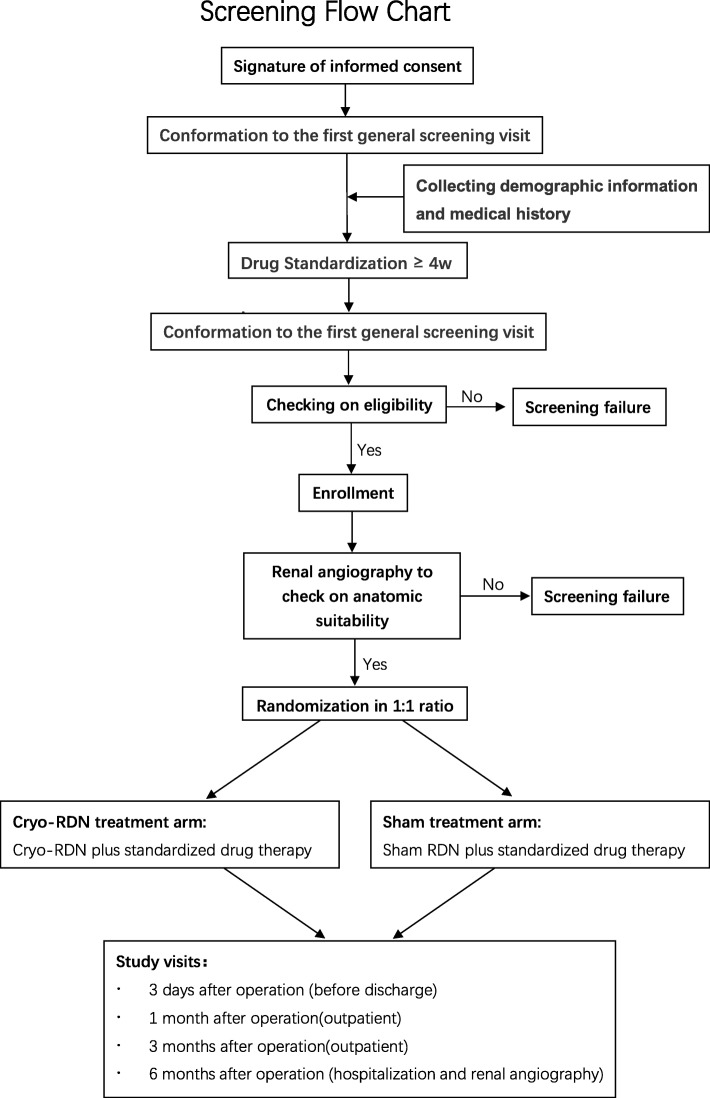
Screening flow chart. 4w 4 weeks, cryo-RDN cryoablation renal denervation

Our trial is a prospective multicentered single-blinded randomized sham-controlled trial to evaluate the efficiency in BP reduction and safety in Chinese patients with uncontrolled hypertension compared with the sham arm. It uses a Cryofocus RDN device. The study will be conducted in accordance with the principles of the Declaration of Helsinki in 13 medical centers nationwide. Each patient will be screened twice before receiving the cryo-RDN, if applicable. A brief flow chart (Fig. 2) is listed below.

### Study patients

In total, 200 patients with uncontrolled hypertension despite receiving pharmacological therapy from 13 clinical centers in China will be enrolled. The definition of uncontrolled hypertension is given in Inclusion criteria at the first screening visit section. At the first visit, patients will be screened for eligibility. After being fully informed about the study and all risk factors, patients will be asked to sign the informed consent form. They will then be prescribed the standardized medications for no less than 4 weeks as a run-in period. At the second enrollment screening visit, patients who meet the additional inclusion criteria will undergo an additional preintervention examination. Parameters at the second screeing visit will be set as baseline and the intervention has to be performed within 2 week.

#### Inclusion criteria at the first screening visit

Patients must meet all the following criteria:

1. Patient is ≥18 and <75 years old at the time of randomization.
2. Patient has continually taken antihypertensive drugs ≥3 months.
3. Patient agrees to cooperate with all study procedures.
4. Patient provides written informed consent to participate in this clinical study.

In addition, the patient must meet one of the following conditions:

1. Patient is currently on two or three types of antihypertensive drugs with 150 mmHg ≤ office SBP ≤ 180 mmHg and office DBP ≥90 mmHg.
2. Patient is currently on four types of antihypertensive drugs with office SBP ≥140 mmHg and office DBP ≥ 90 mmHg.
3. Patient has been on five types of antihypertensive drugs for ≥3 months, and office SBP ≥140 mmHg, office DBP ≥ 90 mmHg, and 24-h SBP ≥135 mmHg.

#### Exclusion criteria at the first screening visit

Patients will be excluded if they meet any of the following criteria:

1. Patient is diagnosed with secondary hypertension.
2. Patient has a history of renal artery interventional therapy, for example, stents.
3. Patietnts has renal artery disease or the presence of morphology variation (including but not limited to aneurysm, stenosis, severe calcification of the renal artery, dissection, and multiple renal arteries).
4. Patient had a myocardial infarction, unstable angina pectoris, syncope or a cerebrovascular accident within 6 months prior to screening
5. Patient has widespread atherosclerosis with recorded intravascular thrombosis.
6. Patient has heart valve disease with significant hemodynamic changes.
7. Patient has uncontrolled pulmonary hypertension.
8. Patient has an estimated glomerular filtration rate of less than 60 mL/min/1.73 m2 according to the Modification of Diet in Renal Disease formula.
9. Patient has a tendency for significant bleeding or blood system disease (platelet count <50 × 10^9^/L or INR>1.5).
10. Patient has an implantable cardioverter defibrillator or a pacemaker.
11. Patient has a severe or systemic infection.
12. Patient has type 1 diabetes mellitus.
13. Patient has a condition, except sleep apnea hypopnea syndrome, that requires long-term oxygen inhalation or mechanical ventilation.
14. Patient is allergic to the contrast.
15. Patient is pregnant, breastfeeding, or preparing for pregnancy.
16. Patient is addicted to alcohol or other drugs.
17. Patient has been diagnosed with a mental illness.
18. Patient has any other disease that may have an adverse effect on their safety in the context of the trial.
19. Patient has been enrolled in any other clinical trial of a drug or device within 6 months prior to enrollment.
20. Patient has any other condition that makes their participation inappropriate, at the investigator’s discretion.

#### Inclusion criteria at the second screening visit

The participant must meet one of the following conditions:

1. 150 mmHg ≤ office SBP < 180 mmHg, office DBP ≥90 mmHg, and 140 mmHg ≤ 24-h SBP < 170 mmHg: Patient is suitable for two or three types of drugs. They will be instructed to take the standardized pharmacological therapy for at least 4 weeks (Additional file 1 and Additional file 2: Table S1).
2. Office SBP ≥140 mmHg, office DBP ≥90 mmHg, and 24-h SBP ≥135 mmHg: Patient is suitable for four types of drugs. They will be instructed to take the standardized pharmacological therapy for at least 4 weeks.
3. Patient is currently on five types of antihypertensive drugs: They will continue to use these drugs until the end of follow-up.

#### Withdrawal

Patients may be withdrawn from the study for any of the following reasons:

1. They may choose to withdraw for any reason.
2. They have severe side effects that require the use of test product to be stopped during the intervention.
3. Based on the investigator’s discretion, the patient is no longer eligible for the study for any reason.
4. Any other rational reason to withdraw them.

### Randomization

A total of 200 enrolled patients will be scheduled for renal angiograms to confirm the anatomical suitability of their renal arteries. A stratified block randomization of patients to receive cryo-RDN or the sham procedure will be performed at a ratio of 1:1 according to study site. To minimize the human factors, the allocation will remain concealed until the patients are anesthetized in the interventional lab. In addition, patients allocated to the sham group will be anesthetized for 30 min in the same manner as those undergoing cryo-RDN.

### Interventions

Eligible patients will undergo either cryo-RDN with Cryofocus system or dual renal angiography only. It is expected that their previously prescribed standardized antihypertensive drugs will remain unchanged in the 6-month follow-up period after the intervention.

However, if in the first 3 months after the intervention a patient experiences dizziness, fatigue, or other symptoms of hypotension, or after 4–6 months the patient has office SBP < 100 mmHg and office DBP<50 mmHg, then the investigators can decrease the number or the dosage of the antihypertensive drugs. Moreover, additional drugs can be added at 4–6 months if a patient taking two or three types of standard antihypertensive drugs has office BP>160/110 mmHg or if a patient taking four types of standard antihypertensive drugs has office BP > 180/110 mmHg (the rules for adjusting dosages are listed in Additional file 2: Table S2).

### Cryo-RDN procedure

Angiography and cryo-RDN procedures will be performed through the femoral artery by one or two designated interventionists at each study site after a combined training session. Under general anesthesia, a 10F sheath and a 6F Judkins Right (JR4.0) catheter will be inserted through femoral access into the renal arteries, and angiography will be performed on dual renal arteries to confirm anatomic feasibility and ablation sites. Intraprocedural anticoagulation will be administered with 100 units of heparin per kg. After randomization based on computer-generated random numbers, patients in the sham arm will be maintained under anesthesia for another 30 min. For patients in the cryo-RDN arm, the 6F catheter will be removed and, according to angiography, properly sized cryoablation balloons will be advanced to the distal segment of the main renal artery through an 8F guidance catheter and a 0.018″ SV-5 guide wire (Cordis, Johnson & Johnson, North West, Florida, USA). During the cryoablation procedure, the regional renal blood flow will be blocked for approximately 5 min on each side with cooling (average −95 °C, ranging between –115 and –80 °C) and rewarming. If needed, the cryoablation balloon catheter will be withdrawn and placed in a catheter of the right size for ablation on the other side of the renal artery. In addition, a bifurcation with a diameter ≥3.0 mm will also be additionally ablated, as a recent expert consensus suggests that this gives better clinical outcomes [7]. A final renal angiography will check that there is no renal artery obstruction, perforation, or dissection. The delivery system and sheath will be removed. Hemostasis of the femoral access site will be achieved using standard techniques.

### Study visits

A total of four visits will be scheduled for participants after the intervention, which will occur before hospital discharge and at 1, 3, and 6 months after discharge. At baseline and at all four visits, a clinical and biochemical evaluation will be undertaken and office BP and 24-h ambulatory BP will be measured. Another renal angiography will occur at the 6-month follow-up. At the 1-month visit, patients will be considered as outpatients and informed of their subsequent drug prescription. The other two follow-ups will require hospitalization.

### Outcome measures

The primary outcome of this study is the difference in the change in 24-h SBP from baseline to 6 months between the cryo-RDN treatment arm and the sham treatment arm. At each participating site, one or two staff members from the laboratory department, and not the clinical department, will be allocated to undertake the 24-h BP measurements during the study period.

The secondary outcomes are as follows:

- Difference in change in 24-h SBP (daytime and nighttime) from baseline to 6 months between the cryo-RDN treatment arm and the sham treatment arm
- Difference in change in 24-h SBP (daytime, nighttime, and 24-h) from baseline to 6 months between the cryo-RDN treatment arm and the sham treatment arm
- Difference in change in office BP (SBP and DBP) from baseline to 6 months between the cryo-RDN treatment arm and the sham treatment arm
- Characteristics of patients who required an adjustment to their medication after the intervention
- Adherence of patients to taking their antihypertensive drug based on the eight-item Morisky Medication Adherence Scale
- Operational performance evaluation of the test device in the cryo-RDN treatment arm
- Side effects possibly caused by the test device
- Major adverse events
- Other adverse events

Safety endpoints primarily include any adverse incidents, including but not limited to puncture-related vascular injury, renal artery complications, renal complications, systematic reactions and a change in renal function, which may be caused by the test device or other factors.

### Sample size calculation

PASS 13 was used to estimate the sample size. This trial was designed to compare the difference in 24-h SBP as a change from baseline to 6 months between the cryo-RDN treatment arm and the sham treatment arm. Based on data from previous studies, we chose a sample size of 81 per treatment arm. The between-group comparison will be powered at 95% to establish the superiority of cryo-RDN for the primary endpoint at a single-sided significance level of 0.025, considering a true 24-h SBP difference as 8 mmHg with a common standard deviation of 18 mmHg. Given an estimated dropout rate of approximately 20%, we increased the sample size to 100 per arm for a total of 200 participants.

### Statistical analysis

The statistical analysis plan will be developed and performed by independent statisticians from the data monitoring committee. SAS software V.9.4 (SAS Institute, Cary, NC) will be used for the statistical analysis. Based on the intention-to-treat principle, the full analysis set and per protocol set will be adopted as the primary analysis populations. A two-sided *p* < 0.05 will indicate significance in all statistical tests. Appropriate descriptive indicators and hypothesis testing methods will be selected cautiously. Data, including demographics, baseline characteristics, and safety, will be summarized by treatment arm.

The primary efficiency outcomes are powered by a superiority test to detect the change in 24-h SBP from baseline to 6 months after the intervention. The sensitivity analysis will have a stratifying variable center as a fixed effect of an analysis of covariance (ANCOVA). A BP reduction will be tested by paired *t*-tests with 95% confidence intervals for the differences between treatment arms. Other categorical data will be tested using either Pearson’s χ^2^ test or Fisher’s exact test. Rank data will be tested by either the Wilcoxon rank sum test or the Cochran–Mantel–Haenszel test. Mixed-model repeated measures analysis including terms for the baseline measurement, types of drugs taken, and medication adjustments during the follow-up will be used for the reduction in BP.

## Discussion

This study was designed to assess the safety and efficacy of cryo-RDN using Cryofocus balloon catheters in patients taking polypharmacy with uncontrolled hypertension.

The short-term safety of this cryo-RDN device has been verified in healthy swine. Plasma norepinephrine and RAS (renin angiotensin system) levels were blunted, and SBP (systolic blood pressure) had declined 28 days after cryoablation. No major restenosis occurred in the renal artery, and the sympathetic nerves peripheral to the renal artery exhibited necrosis and perineurial fibrosis [8]. In addition, recent results from the First-in-man study also showed that the mean ambultory blood pressure reduction was 11.8/8.0 mmHg at the 6-month follow-up of six patients with uncontrolled hypertension. Based on these results, we focused on the safety and efficacy in the longer term for a larger population. RDN has only been carried out internationally for 10 years and large clinical studies of this device-based method have had inconsistent results. So far, the longest trial follow-up was 36 months in HTN-1 and HTN-2. The average office SBP reduction was 21–33.6 mmHg and there was a 53–85% response rate, demonstrating the long-term sustainability of RDN in BP reduction [11, 12]. The global boom in RDN research suffered a setback with the negative results from HTN-3 [13, 14]. However, poor drug adherence, isolated systolic hypertensive patients, and inexperienced operators may have distorted the results [15, 16]. A more rigorous study design is needed. As recommended by European Society of Hypertension, ambulatory BP is of more prognostic value than office BP, which may be confounded by white coat hypertension and other factors [17]. The positive results of HTN-OFF MED have again validated the effectiveness of RDN regardless of which antihypertensive drugs are taken [7]. Results from HTN-OFF MED, HTN-ON MED, and Radiance SOLO were published this year. These trials had a 24-h SBP primary endpoint. BP reduced by an equivalent of less than 10 mmHg. Thus, the antihypertensive effect and safety of RDN are widely accepted [6, 18].

Statistics have shown that the hypertension control rate in China was only 13.8% in 2012 [10]. This low rate is not satisfactory and there are limitations to drug treatment.

The following conclusions can be drawn:

1. The antihypertensive effect of RDN can be maintained for days, and there appears to be no fluctuations due to metabolic effects unlike pharmacological therapy.
2. The antihypertensive effect of RDN is effective long term and does not depend on drug adherence.
3. The antihypertensive effect of RDN does not conflict with drug action, and it is not offset by whether patients are taking medication.

However, to our knowledge, almost all large clinical studies have used individuals from Europe and America as research objects. No trial published so far includes China. In addition, most RDN studies are radiofrequency-based or ultrasound-based. To date, there was only one study reported great antihypertensive effect of cryoablation RDN on non-responders to radiofrequency ablation. But that was using atrial fribrillation ablation catheter as a remedy. [3]. Clinically, cryoablation has been widely used in therapies for cardiac disease and tumors [19, 20]. The formation of intra- and extracellular ice crystals and vascular stasis develop shortly after thawing, which results in cell and tissue death. Thus, cryoenergy was adapted to destroy hyperactivated sympathetic nerves. Previous animal experiments have revealed that (1) cryoablation is more penetrating than radio waves, resulting in fewer (neurofilament light)-positive nerves remaining [21, 22], (2) cryoenergy is more protective and friendlier to peripheral tissues than radio waves in arrythmia ablation [23–26], (3) radiofrequency catheters ablate lesions circumferentially but the lesions vary in shape, area, and diameter depending on the adjacent tissue substructures. Based on these potential advantages, we constructed a special cryoablation balloon with a spherical ablation pattern.

Therefore, we will focus on the safety and efficacy of cryo-RDN in the Chinese population. The aim is to provide a new way to improve the control of hypertension in China as a complement to drug therapy.

According to the latest recommendations, our study uses 24-h ambulatory BP as the primary endpoint and office BP as the secondary endpoint. Because RDN is cost-effective, we can treat a range of participants from 18 to 80 years old [27]. RDN was first used for patients with resistant hypertension. In our study, enrolled patients have 24-h SBP ≥135 mmHg and 24 h-h DBP ≥90 mmHg and are taking two to five types of hypertensive drugs, which qualifies these individuals as mild to moderate combined hypertensive patients. Thus, we have potentially expanded the target population for cryo-RDN. However, a relatively higher drug adherence is expected, as less discomfort and lower BP fluctuations will be observed for these groups of patients. Drug adherence and patient compliance are generally poor for this chronic cardiovascular disease. Approximately 50% of hypertensive subjects in RDN studies are defined as non-adherent by biochemical assay, and 3–5% of these individuals never take their prescribed medicines [28, 29]. Hence, antihypertensive drugs will be prescribed to patients at every follow-up. Further details about the study is provided in Additional files 2.

### Trial status

Recruitment had not yet begun when this paper was submitted.

## Additional files

Additional file 1: Tables for drug standardization. (DOCX 16 kb)

Additional file 2: SPIRIT 2013 checklist: recommended items to address in a clinical trial protocol and related documents. (DOCX 40 kb)

## Data Availability

The datasets generated and analyzed during the current study will be available from the corresponding author on reasonable request.

## Abbreviations

ABPM: 24-h ambulatory blood pressure monitor
ANCOVA: Analysis of covariance
BP: Blood pressure
cryo-RDN: Cryoablation RDN
DBP: Diastolic blood pressure
RDN: Renal denervation
SBP: Systolic blood pressure
