# Correction to: Efficiency and safety of renal denervation via cryoablation (Cryo-RDN) in Chinese patients with uncontrolled hypertension: study protocol for a randomized controlled trial

**DOI:** 10.1186/s13063-019-3979-y

**Published:** 2019-12-27

**Authors:** Han Chen, Meng Ji, Yi Zhang, Yawei Xu, Lingjuan Qiao, Li Shen, Junbo Ge

**Affiliations:** 1 Department of Cardiology, Zhongshan Hospital, Fudan University, Shanghai, China; 2 Institute of Biomedical Sciences, Fudan University, Shanghai, China; 3 Department of Cardiology, Shanghai Tenth People’s Hospital, Shanghai, China; 4 CryoFocus MedTech (Shanghai) Co., Ltd., Shanghai, China; 5 Shanghai Institute of Cardiovascular Diseases, Shanghai, China

**Correction to: Trials (2019) 20:653**

**https://doi.org/10.1186/s13063-019-3693-9**

After publication of our article [1] we were notified that the word “References” was wrongly included in the title.

Originally published title:

- ReferencesEfficiency and safety of renal denervation via cryoablation (Cryo-RDN) in Chinese patients with uncontrolled hypertension: study protocol for a randomized controlled trial

Correct title:

- Efficiency and safety of renal denervation via cryoablation (Cryo-RDN) in Chinese patients with uncontrolled hypertension: study protocol for a randomized controlled trial

The original article has been corrected.
