# Supplementary material for: ReferencesEfficiency and safety of renal denervation via cryoablation (Cryo-RDN) in Chinese patients with uncontrolled hypertension: study protocol for a randomized controlled trial
Source: Trials. 2019 Nov 28;20:653. doi: 10.1186/s13063-019-3693-9 (PMC6883652; doi:10.1186/s13063-019-3693-9)
Supplement: Supplementary file 1 — Tables for drug standardization. (DOCX 16 kb) [file 13063_2019_3693_MOESM1_ESM.docx]

**Supplement Table 1.**

**Antihypertensive drugs standardization**

**Table 1-1. For patients currently on 2 types of drugs**

|  | **Drug type** | **Drug name** | **Dosage** | **Usage** | **Manufacturer** |
| --- | --- | --- | --- | --- | --- |
| **1** | CCB | Amlodipine Besylate Tablet | 5mg | 5mg, qd | Pfizer pharmaceutical Co., Ltd. |
| **2** | ARB | Diovan | 80mg | 80mg, qd | Novartis pharmaceutical Co., Ltd. |

**Table 1-2. For patients currently on 3 types of drugs**

|  | **Drug type** | **Drug name** | **Dosage** | **Usage** | **Manufacturer** |
| --- | --- | --- | --- | --- | --- |
| **1** | CCB | Amlodipine Besylate Tablet | 5mg | 5mg, qd | Pfizer pharmaceutical co., Ltd. |
| **2** | ARB | Co-Diovan | 80mg | Combination drug, 80mg /12.5mg, qd | Novartis pharmaceutical Co., Ltd. |
|  | Diuretic |  | 12.5mg |  |  |

**Table 1-3. For patients currently on 4 types of drugs**

|  | **Drug type** | **Drug name** | **Dosage** | **Usage** | **Manufacturer** |
| --- | --- | --- | --- | --- | --- |
| **1** | CCB | Amlodipine Besylate Tablet | 5mg | 5mg, qd | Pfizer pharmaceutical Co., Ltd. |
| **2** | ARB | Co-Diovan | 80mg | Combination drug, 80mg /12.5mg, qd | Novartis pharmaceutical Co., Ltd. |
|  | Diuretic |  | 12.5mg |  |  |
| **3** | Aldosterone receptor antagonist | Spironolactone | 20mg | 20mg, qd | Shanghai Pharmaceutical (Group) Co., Ltd. Xinyi Pharmaceutical General Factory |

**Supplement Table 2.**

**Antihypertensive drugs adjustment**

**Table 2-1. Drug reduction principle**

| Types of current drugs | 1^st^ drug reduction | 2^nd^ drug reduction | 3^rd^ drug reduction |
| --- | --- | --- | --- |
| 5 | Depend on patients’ condition and medication | | |
| 4 | Spironolactone, 1 pill | Replace Co-Diovan with Diovan, 1 pill | Diovan, 1 pill |
| 3 | Replace Co-Diovan with Diovan, 1 pill | Diovan, 1 pill |  |
| 2 | Diovan, 1 pill |  |  |

**Table 2-2. Drug increasing principle**

| Types of current drugs | 1^st^ drug increasing | 2^nd^ drug increasing | 3^rd^ drug increasing |
| --- | --- | --- | --- |
| 2 | Replace Diovan with Co-Diovan, 1 pill | Spironolactone, 1 pill | Depend on patients’ condition |
| 3 | Spironolactone, 1 pill | Depend on patients’ condition |  |
| 4 | Depend on patients’ condition | |  |
| 5 | Depend on patients’ condition and medication | |  |
